# Renal Colic

**Published:** 1894-04

**Authors:** A. Quackenbush

**Affiliations:** Belleville, Ontario, Canada


### RENAL COLIC.

A. Quackenbush, M. D., Belleville, Ontario, Canada

I wish to record my experience of the efficacy of homœopathic treatment, so I am going to write a short article on two cases of renal colič for publication.

I may be considered by some a crank on renal colic, but I heard so much about the inefficiency of Homœopathy in renal colic in college that when I meet a case I stick to the *low* potencies and "hewed to the line," as in all other diseases.

Case 1.—I was called on Saturday, January 13th, about 7 P. M., to see a man suffering with intense pain. He was a strong, robust, massive man, about thirty-five years of age. When I went in he said: "I have my old colic back again. Now for God's sake and my sake do not give me Morphia, as I would rather suffer from pain." I said I shall not give you that.

He was lying on his back and grating his teeth. He could lie on the back by placing his arm across the back so as to get pressure, or lie on *something hard* for *relief*. He could not sit up, but could walk around, which relieves the pains, but sitting upright always aggravated the pains.

The pains ran down the legs, and with each paroxysm his legs would be flexed on the body because of cramps in the flexor muscles of the thigh and legs. The pains seemed to run from the lumbar region down the thighs.

When the paroxysms were absent he felt restless, and the whole body felt as if he had over-exerted himself.

I prescribed Rhus-t.$^{1m}$ one dose and Placebo. In twenty minutes he was asleep, and was comfortable until the next afternoon about six o'clock. I was out when his wife called; but when I got to the office I saw an order on my slate, and started out with a bottle of Rhus-t.$^{cm}$ in my pocket. I had anticipated what the trouble was. When I arrived the usual number of old women had gathered, and were looking as grave as possible. I walked in and found our patient in his former condition, and he repeated his former request, that I should give no Morphia. I gave Rhus$^{cm}$, and in twenty-two minutes by the watch he was snoring soundly.

Now if this were a mere coincidence, how did it happen the same way twice in succession?

He had been for days in pain in former attacks, and treated scientifically with Morphia, etc., and would be nearly dead for days afterward.

In a day or two he went to work.

CASE 2.—Was called this morning to see a case. A Frenchman, who could scarcely speak a word of English. I found him walking around with his hand pressed into the left lumbar region, moaning and complaining. I looked him over the best I could, and could find out nothing but pain, and better from being continually on the move. I prescribed Rhus$^{1m}$ with some relief. He then lay down on the bed, and when he would turn on the right side he said something rolled from the left to the right side in the abdomen, and the pains went from the left renal region across the abdomen. The pains coming on severely, and a choking with it, in which he loosened his collar, I gave Lachesis$^{75m}$ with temporary relief. I had to leave for a short time, and when I came back his wife said he felt as if the abdomen was crushed in, and would not let any one come near or touch him, for fear they would hurt him. The pains now being very severe, I prescribed Arnica$^{1m}$, and the relief came on gradually but surely. The pains lasted for about three hours after (but he was comfortable), when they finally subsided. He had been in all about four and a half hours from the onset until it finally subsided.

The lesson we can draw from this to older men is not new; but I am writing this for younger men, who, like myself, have not had the experience in those cases of excruciating pain where the scientific (?) chappies prescribe Morphia, etc.; and if the patient dies the friends are consoled by knowing that their bereaved and departed friend has six feet of ground for peaceable possession, and that the best was done that could be done for the patient.

A lesson that may be noted in the first case is that if we prescribe the remedy called for we will get results. I have been unable to find that Rhus has been given in renal colic as far as I have searched the literature on the subject; but I saw it was needed and gave it. Some may say that two cases prove nothing—no more than one swallow makes a summer; but there are enough homœopaths that have had enough to make the earlier months of the warm season.

One thousand cases would prove nothing to some people, but to others one or two will be appreciated.

If this will be of any use to the beginner I shall deem myself well paid for the time expended in writing this article.

